# Implementation and Evaluation of a Pilot Training to Improve Transgender Competency Among Medical Staff in an Urban Clinic

**DOI:** 10.1089/trgh.2015.0009

**Published:** 2016-01-01

**Authors:** Corina Lelutiu-Weinberger, Paula Pollard-Thomas, William Pagano, Nathan Levitt, Evelyn I. Lopez, Sarit A. Golub, Asa E. Radix

**Affiliations:** ^1^ Department of Psychology, Hunter College of the City University of New York, New York.; ^2^ NYU Lutheran Caribbean American Family Health Center, Brooklyn, New York.; ^3^ NYU Lutheran Family Health Centers, Brooklyn, New York.; ^4^ Callen-Lorde Community Health Center, New York.

**Keywords:** competency training, evaluation, health disparities, transgender

## Abstract

**Purpose:** Transgender individuals (TGI), who identify their gender as different from their sex assigned at birth, continue facing widespread discrimination and mistreatment within the healthcare system. Providers often lack expertise in adequate transgender (TG) care due to limited specialized training. In response to these inadequacies, and to increase evidence-based interventions effecting TG-affirmative healthcare, we implemented and evaluated a structural-level intervention in the form of a comprehensive Provider Training Program (PTP) in TG health within a New York City-based outpatient clinic serving primarily individuals of color and of low socioeconomic status. This pilot intervention aimed to increase medical staff knowledge of TG health and needs, and to support positive attitudes toward TGI.

**Methods:** Three 2-h training sessions were delivered to 35 clinic staff across 4 months by two of the authors experienced in TG competency training; the training sessions included TG-related identity and barriers to healthcare issues, TG-specialized care, and creating TG-affirmative environments, medical forms, and billing procedures. We evaluated changes through pre-post intervention surveys by trainees.

**Results:** Compared to pre-training scores, post-training scores indicated significant (1) decreases in negative attitudes toward TGI and increases in TG-related clinical skills, (2) increases in staff's awareness of transphobic practices, and (3) increases in self-reported readiness to serve TGI. The clinic increased its representation of general LGBT-related images in the waiting areas, and the staff provided highly positive training evaluations.

**Conclusion:** This PTP in TG health shows promise in leading to changes in provider attitudes and competence, as well as clinic systems, especially with its incorporation in continuing education endeavors, which can, in turn, contribute to health disparities reductions among TG groups.

## Introduction

Despite increasing awareness of their extreme marginalization, transgender individuals (TGI), who identify their gender as different from their sex assigned at birth,^1–4^ continue to face widespread discrimination, maltreatment, and ostracism,^5,6^ including in social and healthcare services.^7–9^ Providers often lack expertise in adequate transgender (TG) care due to limited specialized training.^10^ A survey of 150 US and Canadian medical schools found that the median total time dedicated to LGBT-related content was 5 h, with zero instruction hours during clinical years. The least represented areas of the curriculum regarding TGI were aspects related to medical and surgical transition care.^10^

In addition, many social service and medical providers hold transphobic beliefs that result in denial of services, and verbal and physical abuse directed toward TG patients.^6,8^ Consequently, TGI evidence low rates of healthcare utilization (HCU)^6,11^ and increased morbidity and mortality than other groups, especially evident among TGI of color.^6,8^

Hostile service-provision environments and lack of cultural competence have been linked to TGI suboptimal care^11,12^ and elevated risk behaviors.^13^ In a national survey (*N*=6450) examining experiences of TGIs including in the healthcare domain, 19% were refused medical care due to their TG identity, 50% had to teach their providers about TG issues, and nearly a third (28%) postponed seeking care due to anticipated discrimination or financial difficulties (48%). TGIs of color experience most discrimination.^6,14–16^

Awareness of TGIs' unique syndemics,^17,18^ and healthcare barriers has been increasing,^1,19^ as has the need for multilevel interventions to improve healthcare access.^12,13,15,18,20^ While individual-level interventions are being investigated,^11,21^ structural-level interventions are less likely to be implemented and evaluated.^6,12,22^ Without systemic changes that facilitate culturally competent and medically appropriate healthcare access, sustainable and effective changes will not occur.^22^

The absence of TG representation in health professions training and their effectual exclusion from mainstream health services have impeded the development of systems and expertise that can accommodate their needs, requiring an overhaul of existing structures.^6,23^ Healthcare systems are organized around dichotomous gender categories,^5^ resulting in unique challenges in addressing the specific needs of TG persons and removing barriers to care.^1,6^

Examples of health system level barriers include the challenges associated with ordering tests that appear to be discordant with a person's affirmed gender, for example, obtaining a cervical pap smear for a TG male or prostate-related tests for a TG female. Other problems can arise with billing systems that reject codes on the basis of perceived “incorrect” gender, or insurance companies that may reject claims based on apparent gender discordance. Other barriers to care include refusal of commercial insurance to routinely cover gender-affirming services, and the designation of TG persons as having “gender identity disorder,” which is regarded by many community members and healthcare workers as pathologizing.^6,7,24–27^ The barriers most cited about clinical experience with TG patients are related to inadequate knowledge about the general and transition-related health needs of TG clients.^28^

In response to the lack of evidence-based interventions to improve the provision of culturally appropriate healthcare to TG clients,^19^ we teamed with a New York City-based outpatient health center serving primarily individuals of color and of low socioeconomic status, to implement and evaluate a structural-level intervention in the form of a comprehensive Provider Training Program (PTP) in TG health. The goals of this pilot intervention were to increase provider knowledge of TG health and needs, and to support positive attitudes toward TGI. As such, we evaluated (1) the acceptability and feasibility of the PTP delivered to all clinic staff and (2) the preliminary efficacy of the PTP in (a) improving provider TG knowledge and attitudes and (b) creating a TG welcoming environment at the clinic. Moreover, we aimed to contribute program evaluation data, given that there are no current formal evaluations of similar existing programs around the country, although the evaluation component of such endeavors is essential in establishing their potential to effect change.

## Methods

### Participants

To pilot test our PTP intervention, we partnered with the New York University Lutheran Family Health Centers (LFHC) in New York City. LFHC provides comprehensive healthcare to a racially diverse and low-income patient population, and it has recently begun serving TGI in an area in which more TG services are needed. One of the authors was contacted by LFHC to provide this type of training to their center (given his expertise in TG medical care and needs), and we worked with the administration to establish the structure of the sessions (total of three, as described hereunder), as well as the evaluation component (pre- and post-training trainee surveys, assessment of the physical environment, and trainee evaluation of their experience, as described in the Evaluative Components section). We invited staff at all levels (from security officer to billing staff) to participate in the training, the rationale for this being that TGI in the United States have reported encountering resistance and/or discrimination at every step of the HCU process.^6^ The trainees included physicians, registrars, nurses, program staff (e.g., prevention counselors), social service providers, patient coordinators, administrative staff, security guard, and billing staff. All staff present on training days participated in the training sessions. The LFHC and NYU Lutheran Medical Center (NYULMC) have recently incorporated the implementation of changes to achieve LGB- and especially TG-affirmative practices in their mission. Although participation in the evaluation component of the study was voluntary, training participation was part of LFHC's mandatory staff meetings/professional development. In addition, given the brief pilot nature of the project, makeup sessions were not possible to implement. Informed consent to participate in the evaluation survey was obtained from 33 of 35 LFHC staff members who attended the training sessions. This study was approved by both the Hunter College and LFHC Institutional Review Boards.

### Training structure and content

Between March 5 and July 11, 2015, we delivered three separate training sessions, based on the main content components of the PTP. Training scheduling revolved around staff availability, given this busy clinic in an urban setting, and took place during administrative hours. Each training session lasted 2 h, thus amounting to a 6-h long training program. The general format of the training was didactic, with practice-based example scenarios being provided by the trainers based on their direct TG healthcare experience, and trainee–trainer group format dialogue stemming from these scenarios and trainees' questions.

This curriculum^29–32^ was developed for more than 8 years by the two trainers, who are of TG experience themselves. It has been delivered, in different configurations and durations (based on audiences and their needs), to hundreds of organizations, from educational institutions to direct services providers. However, this current project constitutes its first formal evaluation.

The first training was delivered to all level staff and included terminology, issues of TG identity, stressors, health disparities, the process of social, medical, and surgical “transitioning,” and strategies that can be adopted by medical practitioners and staff to be sensitive and TG affirmative, including the use of appropriate terminology and forms of address across all interactions, from the front desk to HIV-related visits, or cancer screenings. This training was provided by one of the authors, a nurse practitioner, who has 15 years of experience in delivering such curricula across institutions nationally.

The second training was provided by another one of the authors who is a physician with 20 years of experience in both TG healthcare practice and training medical providers to deliver primary care and transition-related care to TGI. This second training was for prescribing providers and covered the current guidelines related to medical transition care, including hormone therapy and long-term monitoring. Finally, the third training was provided by the nurse practitioner trainer, and focused on TG-affirming forms and medical billing that are inclusive of TG identities, to acknowledge their identities and facilitate navigation of insurance billing and coverage. This third training included nonprescribing providers, specifically intake registration staff (the first point of contact for TG patients) and billing staff. As such, each LFHC staff received a total of 4 h of training, with the first 2 h covering general knowledge necessary for all staff to understand TG identities, needs, disparities in, and barriers to healthcare, and the last 2 h consisting of specialized knowledge based on each staff's role at the clinic.

### Evaluation components

We measured outcomes of interest (described hereunder) at two points: before the first training (baseline) and once ∼3 months after the first training (follow-up). We aimed to record changes at two levels: (1) staff level, that is, TG knowledge and attitudes, and satisfaction with the trainings and (2) environmental level, that is, creating a TG welcoming environment at the clinic. All three training sessions included concepts related to expected level 1 staff-level changes, whereas training sessions 1 and 3 addressed level 2 environmental changes. All measures appeared in both baseline and follow-up surveys, except for acceptability measures that were completed by staff immediately after each training session.

### Level 1: staff measures

#### Knowledge and attitudes

For the first category of outcomes, we distributed self-administered paper and pencil surveys to each trainee, which included measures intended to capture changes in TG-related knowledge and attitudes. We used the Sexual Orientation Provider Competency Scale^33^ (alpha=0.90) (adapted for TG competencies), a five-point Likert scale with response options from “strongly disagree” to “strongly agree.” The scale measures self-perceived abilities, attitudes, and knowledge related to TG healthcare. It contains three subscales: *skills* (8 items; alpha=0.91; e.g., “I have received adequate clinical training and supervision to counsel transgender clients” or “I feel competent to assess the health care needs of a person who is transgender”), *attitudes* (10 items; alpha=0.88; for example, “The lifestyle of a transgender individual is unnatural” or “When it comes to transsexuality, I agree with the statement: ‘You should love the sinner but hate or condemn the sin’”), and *knowledge* of the impact of gender normativity on TG patients (8 items; alpha=0.76; for example, “I am aware that medical providers frequently impose their values concerning sexuality upon transgender patients”; “There are different psychological/social issues impacting gays versus lesbians versus transgender individuals”).

To measure attitudes toward TGI, we used the Attitudes toward Transgender Patients Scale^34^ (13 items; adapted for TG issues), with a five-point Likert scale response configuration (from “strongly agree” to “strongly disagree”). Example items are “It is more challenging to discuss sexual behavior with transgender patients than with other patients”; “Transgender patients deserve the same level of quality care from medical institutions as non-transgender patients.” Awareness of providers' transphobic clinical practices was measured through the Clinical Skills and Attitudes Scale^34^ (four items; adapted for TG issues), with a five-point Likert scale response configuration (from “never” to “always”). Example items are “How often have you heard of other providers treating transgender patients differently than non-transgender patients with respect to the following: Less eye contact; Spent most visits screening for STDs.”

We measured endorsement of transphobic attitudes with the Modern Homophobia Scale^35^ (12 items; alpha=0.93; adapted for TG issues), which is a five-point Likert scale with response options between “strongly disagree” and “strongly agree,” and items such as “Transgender individuals still need to protest for equal rights” or “The notion of universities providing students with undergraduate degrees in Gay and Lesbian Studies is ridiculous.” We measured the trainees' readiness to interact with or provide care to TGI by adapting the Contemplation Ladder.^36,37^ The ladder entailed 10 items (configured as rungs on a ladder; alpha values for the precontemplation, contemplation, and action subscales are 0.30, 0.52, and 0.76^38^) pertaining to how ready one was to interact with or provide care to TGI. For example, participants would select number 1 to indicate no intentions to change their comfort level or readiness to provide services to TGI (e.g., “I don't feel comfortable interacting with or providing care to transgender individuals and do not intend to make any changes to that.”); a score of 10 indicates that a change in serving TGI with competence has occurred and it is believed to be permanent (e.g., “I am comfortable interacting with or providing care to transgender individuals and am confident that I will continue feeling this way and showing it through my interactions with and care of them”).

Finally, we included a section on trainee demographics, such as age, racial/ethnic background, income, position at the clinic, previous experience providing care to TGI, and TG-related trainings.

#### Acceptability measures

After each training session, all participants were invited to provide an anonymous evaluation of the training content and instructor. Example questions are: “How helpful was the session in developing a better understanding of transgender identity?”; “How helpful was the session in preparing you (further) in interacting with and caring for transgender individuals?”; or “How knowledgeable do you feel the trainers were?” Response options were on a five-point Likert scale ranging from “not helpful” to “very helpful.” A free text area was also provided to record trainee recommendations for improvement by asking the following two questions: “Did we miss anything and, if so, what would you have liked added?” and “Would you advise us to do things differently and, if so, how?”

### Level 2: clinic environment surveillance measures

To measure possible clinic environment changes related to the training, one of the authors observed the waiting areas and examination rooms both before and after the training. She checked a list of indicators of a TG-welcoming physical environment at the clinic, which included (1) staff wearing a rainbow symbol on their badges, (2) any TG-related health pamphlets or magazines, and (3) TG-related materials displayed on the walls.

### Data analysis

Descriptive statistics were obtained for demographic variables. Before computing scale scores, necessary items were reverse coded. To detect possible changes from baseline to follow-up in our outcomes of interest, we conducted bivariate analyses in the form of nonparametric tests (Wilcoxon signed rank tests), given the pilot nature of this study, and, therefore, its small sample. Significance level was set at *p*<0.05; however, we report differences if the *p* value was below 0.20, because differences may indicate trends of change in the desired direction, which could become significant in a larger sample. Analyses were conducted by using SPSS software 22 (IBM SPSS Statistics, IBM Corporation, Chicago, IL).

## Results

### Feasibility of the training and evaluation

Of the 35 individuals present at the training, two individuals refused to participate in the evaluation component. Of the 33 individuals consenting to participate in the evaluation (94% of all attendees), one baseline evaluation was mislabeled, resulting in us not being able to link their baseline data to their follow-up evaluation data. A total of 32 participants provided evaluation baseline evaluation data we could use in analyses (91% of all attendees).

At the 3-month follow-up, 26 individuals completed the second evaluation (81% of all attendees who provided baseline evaluation data). Six individuals had left LFHC in-between the baseline and follow-up due to the end of a grant-funded project. As such, voluntary participation in our training was 100%, and retention to the 3-month follow-up was high, and we hypothesize that it would have been closer to 100% had the six staff's employment at LFHC not ended.

### Trainee characteristics

Table 1 provides trainee demographics. Participants' mean age was 39.8 years (SD=12.4, range 21–62), 7% identified as gay, 3% as lesbian, 87% as straight, and 3% as other; 71% identified as female. The sample was ethnically and racially diverse (Table 1). A majority of the trainees identified as Hispanic/Latino (71%), with a third of them (29%) also being multiracial, 19% Black/African American, and 23% white. Fifty seven percent of the trainees had, in the past, provided care/services in various capacities to TGI for an average time of 7.5 years, entailing patient navigation, case management, HIV-related services, mental health, coaching and education, and nursing-related care. Forty five percent of participants had undergone a previous training on sexual and gender minority issues (only three of these participants having received TG-specific trainings), with the majority of them (82%) having done that ∼1 year before the present training (data not shown). The majority (91%) were full-time employees at LFHC.

**Table 1.** **Trainee Demographic Characteristics**

Racial/ethnic identification	*n* (%)
Hispanic or Latino	
Yes	22 (71)
No	9 (29)
Asian	1 (3)
Black/African American	6 (19)
White	7 (23)
Multiracial	9 (29)
Other	9 (29)
Education	
High school/GED	1 (3)
Some college	8 (25)
College degree	13 (41)
Graduate degree	9 (28)
Income	
<$20,000	3 (9)
$20,000–$49,000	16 (50)
$50,000–$79,000	5 (16)
>$80,000	5 (15)
Gender identity	
Female	22 (71)
Male	9 (29)
Sexual identity	
Gay	2 (7)
Lesbian	1 (3)
Heterosexual	27 (87)
Other	1 (3)
Age	
21–29	9 (27)
30–39	5 (15)
40–49	10 (30)
50 and above	7 (21)
Not reported	2 (6)

Not every respondent provided answers to each question, therefore, the *n* for each category varies.

### Skills, attitudes, and knowledge

Table 2 presents the results of the evaluation on our outcomes of interest. From baseline to follow-up, there was a significant increase in participants' mean score for self-perceived skills in working with TG patients (M=20.9 vs. M=29.1; *p*<0.01), and a significant decrease in trainees' negative attitudes toward TG patients (M=19.3 vs. M=17.3; *p*<0.05).

**Table 2.** **Reported TG-Related Knowledge and Attitudes from Baseline to Follow-Up**

	Baseline mean (SD)	Follow-up mean (SD)	Test statistic
Sexual orientation provider competency			
Clinical skills	22.1 (6.7)	28.5 (8.4)	*Z*=−2.9^a^
Negative attitudes	19.6 (7.9)	17.1 (8.4)	Z=−2.3^b^
Knowledge of TG clinical issues	26.0 (6.2)	25.4 (6.3)	*n.s.*
Readiness to provide care to TGI	8.6 (2.3)	9.3 (1.8)	*n.s.*
Awareness of provider transphobia	9.2 (4.0)	14.0 (7.0)	Z=−1.3^c^
Transphobia	25.0 (6.8)	25.2 (7.4)	*n.s.*

^a^ *p*<0.01.

^b^ *p*<0.05.

^c^ *p*<0.18.

TGI, transgender individuals.

Although participants' level of readiness in caring for TG patients did not significantly change from baseline to follow-up, there was an increase in that score in the expected direction (from M=8.6 to M=9.3). At the post-training evaluation, participants' awareness of provider transphobic practices had been raised (M=9.2 vs. M=14.0; *p*<0.18). We did not observe changes in the following aspects: trainees' knowledge of the impact of heteronormative perspectives of providers on TG patients, knowledge of applied TG clinical issues, or transphobia (Table 2).

### Environmental changes

In terms of changes in the environment of the clinic, in addition to the postexposure prophylaxis Department of Health posters portraying a TG woman and a gay man that we recorded at baseline, the follow-up environmental surveillance identified three additional elements displayed in the waiting area: PLUS Magazine, Poz Magazine, and a brochure advertising NYULMC services for LGBT patients. Although the latter had been created by NYULMC before the training, it had not been displayed at the baseline surveillance. Although none of these markers are TG specific, they include TGI under the LGBT umbrella. TG-specific materials are not as readily available as are LGBT inclusive markers.

In addition to measures we included in our surveys, during the last training, we worked with staff to take the following steps to increase TG-related inclusion. First, medical forms will be modified to accommodate the chosen name for TGI whose documentation may not be congruent with their gender identity and presentation. Second, staff will adopt a permanent script on how to address all patients when scheduling appointments over the phone and checking in individuals at the front desk (e.g., “How would you like to be referred to?”). This question will also be asked on the forms (of everyone), and this fact will be posted on the wall in the waiting area so that TG patients do not feel singled out (as if this is only asked of them), and such that non-TG patients do not become confused (as to why they are asked this question). Furthermore, staff will adopt a list of TG-affirmative resources and referrals outside of LFHC to be given to TG patients needing such services.

### Acceptability of the training

Lastly, based on the training evaluation surveys we collected at the end of each training session, we obtained 37 anonymous evaluation forms between the three training sessions (Table 3). Ninety-two percent of participants found the training to be very helpful, 87% of participants found the training to be very informative, and 95% of participants found the trainers to be highly knowledgeable.

**Table 3.** **Trainee Session Evaluation Ratings**

	Highly (%)	Moderately (%)
How interested were you?	92	3
How informative was it?	87	8
How knowledgeable were the trainers?	95	5
How helpful were the sessions in….	87	8
..helping you gain more knowledge?		
..helping you feel motivated to make changes in your interactions with transgender individuals?	87	3
..developing you gain a better understanding of transgender identity?	89	3
..preparing you (further) in interacting with and caring for transgender individuals?	84	5

Eighty-seven percent of participants highly agreed that the training helped them gain more knowledge and helped them feel more motivated to make changes in their interactions with TGI. Eighty-nine percent of participants highly agreed that the training helped them develop a better understanding of TG identity and 84% highly agreed that it further prepared them for interactions with TGI.

Qualitative text evaluations provided in the free-writing areas by participants reflected a positive experience, with statements such as “The session was very informative”; “I believe it was a great training and it covered a lot of material. Great information, I liked it!”; and “Everything was very good. Thanking you for being so open and informative, using personal life it always makes it interesting and engaging.”

Participants did make recommendations for additions to the trainings, including more group discussions in smaller groups, more time for questions, extending the time of the training session, role-plays, more interactive activities, and media/visual materials on how to better provide care to TGI, and clinic flow from interactions with security guards to TG-affirmative medical records.

## Discussion

We tested the feasibility, acceptability, and preliminary efficacy of a community-developed PTP, designed to increase sustainable TG health knowledge and cultural competency at all levels of service provision and institutional practice, from front desk to physicians and billing. Delivering such a training within the busy schedule of an urban health center proved to be feasible and highly acceptable by the participants. If adopted and applied routinely within medical practices, this structural training intervention, developed by the TG health expert authors of this article, has the potential to increase sustainable (1) provider knowledge and attitudes toward TGI, (2) TGI service satisfaction, (3) TG-welcoming physical environments, and (4) clinic-level TG care. This pilot, despite being underpowered, identified shifts in trainees' attitudes toward TGI and their care, lending promise to the potential of a larger and improved curriculum (based on these findings) to effect lasting practice-based and institutional changes that remain to be adopted nationwide.^18^

This 6-h pilot training session is specific to TG health issues and comparable in length with the 5-h average medical school education (which diffuses its content across the multiple LGBT groups and is not TG focused). Our training, due to its sole emphasis on TG health, did show immediate positive impact. It is likely, however, that it would have lasting effects with periodic refreshers, which we recommend be included as part of routine institutional staff trainings, alongside with other human resources and clinical practice-based recurring trainings. Our trainees indeed cited the need to have additional sessions beyond the initial 6-h pilot training session, which also points to trainee acceptability and recognition of training needs.

Concerns may exist around the feasibility of implementation of such trainings in real-life settings of service provision (outside of educational settings such as medical, nursing, or social work school, where the primary activity is training). Two of our primary goals in this project were to test the very feasibility of implementing a training program, and, based on this pilot, we proved that the scheduling and execution of delivering three training sessions and two evaluations were highly feasible. Scheduling was iterative, yet, nevertheless, successful, with one instance when we had to reschedule due to staff availability. Second, trainees found the program to be highly acceptable. Based on their ratings, the training was found to be highly acceptable and was received positively by the participants. In addition, they indicated interest in more extensive trainings, with more interactive activities, how to video viewing, role-plays, and case studies. These comments indicate the staff's eagerness for direct TG-affirmative service, which they intend to further master. Trainees viewed the utility of implementing this type of training at an institutional level given that they recommended that all NYULMC staff should benefit from and engage with this type of training, and that TG-affirmative resources should be readily available for use on an as-needed basis. The findings of this evaluation are congruent with the long-term goals of our project to effect lasting structural change.

It is encouraging that a 6-h training curriculum was able to significantly impact trainees' attitudes toward TGI positively and increase their awareness of the nature of transphobic practices, as well as self-reported readiness to serve TGI (even if not significantly so given the small sample). Based on what we learned from this evaluation, repeating exposure to this information in booster modules delivered periodically over time, as well as including role-plays to reach new comfort levels, would likely further increase the impact of the training. Expanding the training based on trainee feedback might lead to changes in aspects that were not impacted by the training, such as trainees' knowledge of the impact of gender-normative perspectives of providers on TG patients or knowledge of applied TG clinical issues. More in-depth clinical practice trainings involving “shadowing” providers specialized in TG care are recommended to address possible gaps in the current PTP structure and increase its impact.

We can note several limitations of this evaluation project. First, due to internal LFHC project funding independent of our evaluation, six individuals were unable to complete the post-training evaluation because their positions at LFHC ended, and LFHC was not able to contact them for the completion of the follow-up survey. This occurrence reduced our initial modest sample size, making it difficult to identify shifts in attitudes and knowledge associated with the training. However, this is to be expected given the pilot nature of this project. In addition, 2 of the 35 LFHC staff declined participation in the evaluation component, which we suspect might have had to do with their lack of trust in the confidentially of their data, despite our strict adherence to human subjects protection guidelines and the surveys being only labeled with numerical identification. Following from the first limitation, second, the certainty of our conclusions would benefit from being strengthened in a future larger trial evaluating the efficacy of this training. Conducting a training at two sites in a wait-list control format and comparing their outcomes would increase the sample size and lend more rigor to this initial evaluation step. Nevertheless, the results of this pilot point to both areas of modification in creating a robust training program, as well as to areas we recommend every similar training program include (e.g., a comprehensive range of topics, such as TG identity and barriers to healthcare besides strictly medical information about hormonal care).^39^

In addition, given the early stages of research regarding TGI, it is difficult to find validated scales; therefore, we resorted to adapting general LGB scales, which may have weakened our construct validity. As this field of research progresses, TG-specific and validated scales should minimize this threat.

Lastly, the fact that approximately half of trainees had prior TG-related experience (training and/or care) may have allowed them to benefit particularly from the training curriculum. Notably, most prior trainings staff experienced were non-TG specific (only three received TG-specific training previously), but rather were included under the larger LGBT umbrella, which does not constitute sufficient preparation for providing care to TGI. In our rationale of selecting a training site for this pilot, we included the fact that the site would be open to caring for TGI, given that effecting these types of changes in a clinic that is hostile to this possibility would be unrealistic. Having this inclusion criterion increased the chance that some of the staff may have already been exposed to similar trainings.

Nevertheless, our evaluation indicates significant movement toward TG competence, even in these nonnaïve trainees, and, therefore, highlights the unequivocal benefit of participation despite prior knowledge. In addition, the staff had not participated in a recent training (not in the previous year and a half), therefore, for those who had, this training acted as a refresher, which we argue is a beneficial model of training. A larger sample would have allowed for adjustment for prior training experience.

Efforts of this nature thus warrant further development and testing, as the need for refining and scaling up these interventions remains high.^18,22,39^ Individual-level stigma-reduction interventions can teach TGI how to cope with stressors at various levels,^21,40^ but do not address the need to create inclusive practices and environments.^18,22^ This PTP structural intervention, which we intend to further fine-tune and test in a wait-list control trial format, can have a significant and sustained impact on TG health by improving accessibility, cultural competence, and quality of healthcare for TGI.^9,12,22,41^

## Abbreviations Used

HCU: healthcare utilization
LFHC: Lutheran Family Health Centers
LMC: Lutheran Medical Center
PTP: Provider Training Program
TG: transgender
TGI: transgender individuals

## References

[1] Centers for Disease Control and Prevention. HIV infection among transgender people: Centers for Disease Control and Prevention, 2011 Available at http:/cdc.gov/hiv/transgender/pdf/transgender.pdf (accessed 1010, 2011)

[2] Burdge BJ Bending gender, ending gender: theoretical foundations for social work practice with the transgender community. Soc Work. 2007;52:243–250 17850032 10.1093/sw/52.3.243

[3] Monro S Theorizing transgender diversity: towards a social model of health. Sex Relat Ther. 2000;15:33–45

[4] Pardo TB Growing Up Transgender: Research and Theory. Cornell University, Family Life Development Center, Ithaca, NY, 2008

[5] Harrison J, Grant J, Herman JL A gender not listed here: genderqueers, gender rebels, and otherwise in the National Transgender Discrimination Survey. J LGBTQ Policy. 2012;2:13–24

[6] Grant JM, Mottet LA, Tanis J, Herman JL, Harrison J, Keisling M National Transgender Discrimination Survey Report on health and health care. Washington, DC: National Center for Transgender Equality and National Gay and Lesbian Task Force, 2010, pp. 1–23

[7] Lombardi E Transgender health: a review and guidance for future research—proceedings from the Summer Institute at the Center for Research on Health and Sexual Orientation, University of Pittsburgh. Int J Transgend. 2011;12:211–229

[8] Bradford J, Reisner SL, Honnold JA, Xavier J Experiences of transgender-related discrimination and implications for health: results from the Virginia Transgender Health Initiative Study. Am J Public Health. 2013;103:1820–1829 23153142 10.2105/AJPH.2012.300796 PMC3780721

[9] Radix A, Lelutiu-Weinberger C, Gamarel K Satisfaction and health care utilization of transgender and gender non-conforming individuals in NYC: a community-based participatory study. LGBT Health. 2014;1:302–308 26789858 10.1089/lgbt.2013.0042

[10] Obedin-Maliver J, Goldsmith ES, Stewart L, White W, Tran E, Brenman S, et al. Lesbian, gay, bisexual, and transgender—related content in undergraduate medical education. JAMA. 2011;306:971–977 21900137 10.1001/jama.2011.1255

[11] Clements-Nolle K, Marx R, Guzman R, Katz M HIV prevalence, risk behaviors, health care use, and mental health status of transgender persons: implications for public health intervention. Am J Public Health. 2001;91:915–921 11392934 10.2105/ajph.91.6.915 PMC1446468

[12] Roberts TK, Fantz CR Barriers to quality health care for the transgender population. Clin Biochem. 2014;47:983–987 24560655 10.1016/j.clinbiochem.2014.02.009

[13] Sugano E, Nemoto T, Operario D The impact of exposure to transphobia on HIV risk behavior in a sample of transgendered women of color in San Francisco. AIDS Behav. 2006;10:217–225 16362237 10.1007/s10461-005-9040-z

[14] Murrill CS, Liu KL, Guilin V, Colon ER, Dean L, Buckley LA, et al. HIV prevalence and associated risk behaviors in New York City's house ball community. Am J Public Health. 2008;98:1074–1080 18445806 10.2105/AJPH.2006.108936 PMC2377289

[15] Nemoto T, Operario D, Keatley J, Han L, Soma T HIV risk behaviors among male-to-female transgender persons of color in San Francisco. Am J Public Health. 2004;94:1193–1199 15226142 10.2105/ajph.94.7.1193 PMC1448420

[16] Koken JA, Bimbi DS, Parsons JT Experiences of familial acceptance—rejection among transwomen of color. J Fam Psychol. 2009;23:85 3 10.1037/a0017198 PMC2840628 20001144

[17] Brennan J, Kuhns LM, Johnson AK, Belzer M, Wilson EC, Garofalo R Syndemic theory and HIV-related risk among young transgender women: the role of multiple, co-occurring health problems and social marginalization. Am J Public Health. 2012;102:1751–1757 22873480 10.2105/AJPH.2011.300433 PMC3416048

[18] Operario D, Nemoto T HIV in transgender communities: syndemic dynamics and a need for multicomponent interventions. JAIDS. 2010;55:S91–S93 21406995 10.1097/QAI.0b013e3181fbc9ec PMC3075534

[19] IOM (Institute of Medicine). The Health of Lesbian, Gay, Bisexual, and Transgender People: Building a Foundation for Better Understanding. Washington, DC: The National Academies Press, 2011 22013611

[20] Herbst JH, Jacobs ED, Finlayson TJ, McKleroy VS, Neumann MS, Crepaz N Estimating HIV prevalence and risk behaviors of transgender persons in the United States: a systematic review. AIDS Behav. 2008;12:1–17 17694429 10.1007/s10461-007-9299-3

[21] Taylor RD, Bimbi DS, Joseph HA, Margolis AD, Parsons JT Girlfriends: evaluation of an HIV-risk reduction intervention for adult transgender women. AIDS Educ Prev. 2011;23:469–478 22010810 10.1521/aeap.2011.23.5.469

[22] Stroumsa D The state of transgender health care: policy, law, and medical frameworks. Am J Public Health. 2014;104:e31–e38 10.2105/AJPH.2013.301789 PMC3953767 24432926

[23] Lee R Topic in review: health care problems of lesbian, gay, bisexual, and transgender patients. West J Med. 2000;172:40 3 10.1136/ewjm.172.6.403 PMC1070935 10854396

[24] Bauer GR, Hammond R, Travers R, Kaay M, Hohenadel KM, Boyce M “I don't think this is theoretical; this is our lives”: how erasure impacts health care for transgender people. J Assoc Nurses in AIDS Care. 2009;20:348–361 19732694 10.1016/j.jana.2009.07.004

[25] Lombardi E Enhancing transgender health care. Am J Public Health. 2001;91:869–872 11392924 10.2105/ajph.91.6.869 PMC1446458

[26] Lombardi E Public health and trans-people: barriers to care and strategies to improve treatment. In: The Health of Sexual Minorities Public Health Perspectives on Lesbian, Gay, Bisexual and Transgender Populations (Meyer I, Northridge M; eds). New York: Springer; 2007, pp. 638–652

[27] Namaste V Invisible Lives: The Erasure of Transsexual and Transgendered People. Chicago, IL: University of Chicago Press, 2000

[28] Snelgrove JW, Jasudavisius AM, Rowe BW, Head EM, Bauer GR “Completely out-at-sea” with “two-gender medicine”: a qualitative analysis of physician-side barriers to providing healthcare for transgender patients. BMC Health Serv Res. 2012;12:11 0 22559234 10.1186/1472-6963-12-110 PMC3464167

[29] Radix A Utilization of health services and health status of transgender patients at a community health center. Paper Presented at the 23rd WPATH Symposium; Bangkok, Thailand 214–18, 2014

[30] Radix A Cardiovascular and bone considerations in patients on cross-sex hormone therapy. Paper Presented at the 23rd WPATH Symposium; Bangkok, Thailand 214–18, 2014

[31] Radix A, Sevelius J, Grant R Care of HIV infected transgender people; HIV chemoprophylaxis (PrEP) in transgender populations. Paper Presented at the National Transgender Health Summit; Oakland, California 417–18, 2015

[32] Radix A, Deutsch M Cardiovascular health; Preventive screening; Bone health; Important recent publications in transgender medicine. Paper Presented at the National Transgender Health Summit; Oakland, California 417–18, 2015

[33] Bidell MP The Sexual Orientation Counselor Competency Scale: assessing attitudes, skills, and knowledge of counselors working with lesbian, gay, and bisexual clients. Couns Educ Superv. 2005;44:267–279

[34] Sanchez NF, Rabatin J, Sanchez JP, Hubbard S, Kalet A Medical students' ability to care for lesbian, gay, bisexual, and transgendered patients. Fam Med. 2006;38:21–27 16378255

[35] Morrison MA, Morrison TG, Franklin R Modern and old-fashioned homonegativity among samples of Canadian and American university students. J Cross Cult Psychol. 2009;40:523–542

[36] Biener L, Abrams D The contemplation ladder: validation of a measure of readiness to consider smoking cessation. Health Psychol. 1991;10:360–365 1935872 10.1037//0278-6133.10.5.360

[37] Slavet J, Stein L, Colby S, Barnett N, Monti P, Golembeske C Jr., et al. The Marijuana Ladder: measuring motivation to change marijuana use in incacertated adolescents. Drug Alcohol Depend. 2006;83:42–48 16289930 10.1016/j.drugalcdep.2005.10.007 PMC2754131

[38] Gavin DR, Sobell LC, Sobell MB Evaluation of the readiness to change questionnaire with problem drinkers in treatment. J Subst Abuse. 1998;10:53–58 9720006 10.1016/s0899-3289(99)80140-0

[39] Goldberg JM Training community-based clinicians in transgender care. Int J Trans. 2006;9:219–231

[40] Garofalo R, Johnson AK, Kuhns LM, Cotten C, Joseph H, Margolis A Life skills: evaluation of a theory-driven behavioral HIV prevention intervention for young transgender women. J Urban Health. 2012;89:419–431 22223033 10.1007/s11524-011-9638-6 PMC3368050

[41] Peitzmeier SM, Reisner SL, Harigopal P, Potter J Female-to-male patients have high prevalence of unsatisfactory Paps compared to non-transgender females: implications for cervical cancer screening. J Gen Intern Med. 2014:1–7 10.1007/s11606-013-2753-1 PMC4000345 24424775

